# Supplementary material for: An On-Demand Drug Delivery System for Control of Epileptiform Seizures
Source: Pharmaceutics. 2022 Feb 21;14(2):468. doi: 10.3390/pharmaceutics14020468 (PMC8879600; doi:10.3390/pharmaceutics14020468)
Supplement: Supplementary file 1 [file pharmaceutics-14-00468-s001.zip › pharmaceutics-1539832-supplementary-final.pdf]

pharmaceutics

Supplementary Materials: An On-Demand Drug Delivery System for Control of Epileptiform Seizures

Takashi Nakano, Shakila B. Rizwan, David M. A. Myint, Jason Gray, Sean M. Mackay, Paul Harris, Christopher G. Perk, Brian I. Hyland, Ruth Empson, Eng Wui Tan, Keshav M. Dani, John NJ Reynolds and Jeffery R. Wickens

Preparation of liposomal nanostructures and hollow gold nano-shells (HGNs)

All chemicals and drugs were purchased from Sigma Aldrich, and phospholipids from Avanti Polar Lipids and Laysan Bio, and used without further purification or modification. Hollow gold nanoshells (HGNs) were prepared as per a previously reported procedure by Prevo and co-workers. [29] HGN suspensions were stabilized to aggregation by surface derivatization with a 750 Da thiol-derived polyethylene glycol at a ratio of approximately 1:7 mmol/mmol, and concentrated by centrifugation at 10000 rcf. All final concentrated HGN suspensions were analysed by inductively-coupled plasma mass spectrometry (ICP-MS) to quantify the total gold concentration in the suspension. The 750 Da thiol-derived polyethylene glycol was synthesized through the reaction of methoxypolyethylene glycol amine (750 Da) with 2-iminothiolane in a 1:2 mol ratio in 3 mM sodium phosphate buffer at pH 9.3. The thiol-derived DSPE-PEG-2000 phospholipid used for nanoparticle conjugation was prepared as previously described, from 1,2-distearoyl-sn-glycero-3-phosphoethanolamine-N-[amino (polyethyleneglycol)-2000] (sodium salt) (DSPE-PEG2000-NH2) using 2-iminothiolane in a 1:2 mol ratio in 3 mM phosphate buffer at pH 9.3. [27]

Liposomal nanostructures were prepared using the thin-film rehydration method as previously described, using a phospholipid composition comprising either 1,2-dipalmitoyl-sn-glycero-3-phosphocholine (DPPC) or 1,2-distearoyl-sn-glycero-3-phosphocholine (DSPC) with cholesterol, sphingomyelin, DSPE-PEG2000, and DSPE-PEG2000-SH in a 100:5:5:4:3.5 mol ratio. [27,28] The lipid components were dissolved in chloroform, combined in the appropriate ratios and the solvent removed in vacuo to form a lipid film. The film was then re-suspended using the appropriate volume of an aqueous solution containing phosphate buffer (20 mM Na2HPO4, Ph 7.4) containing muscimol, or kainic acid. The phospholipid suspension was extruded 15 times through 100 nm polycarbonate membranes to achieve size-controlled unilamellar vesicles. Excess drug was subsequently removed via dialysis. All samples were subjected to DLS analysis after manufacture and before use to characterize size distribution and stability to aggregation. HGN-liposome conjugates were used in experiments generally not more than two weeks after manufacture.

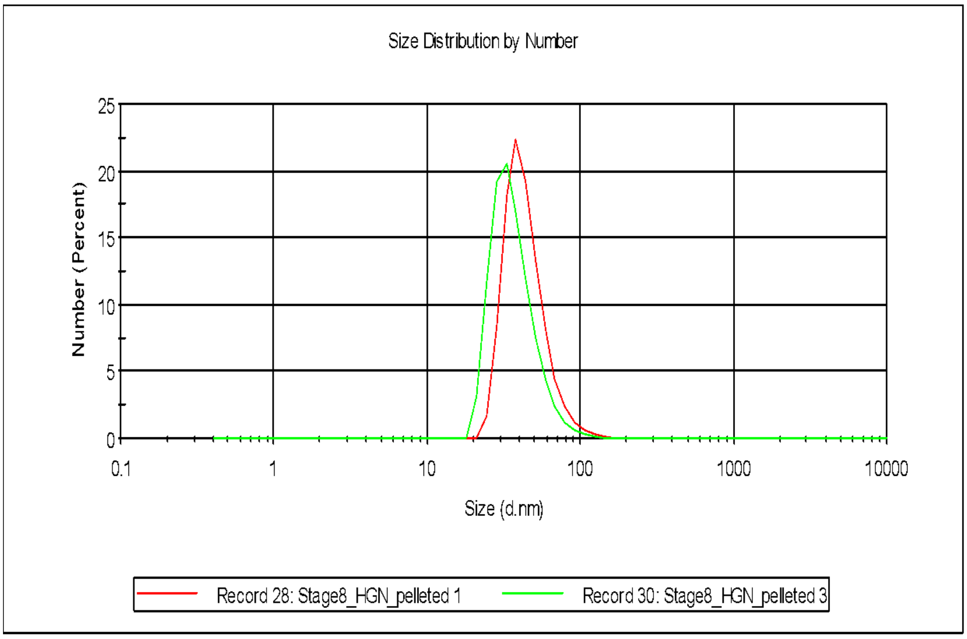

Figure S1. DLS of typical HGN suspensions indicating the hydrodynamic diameter of HGNs.

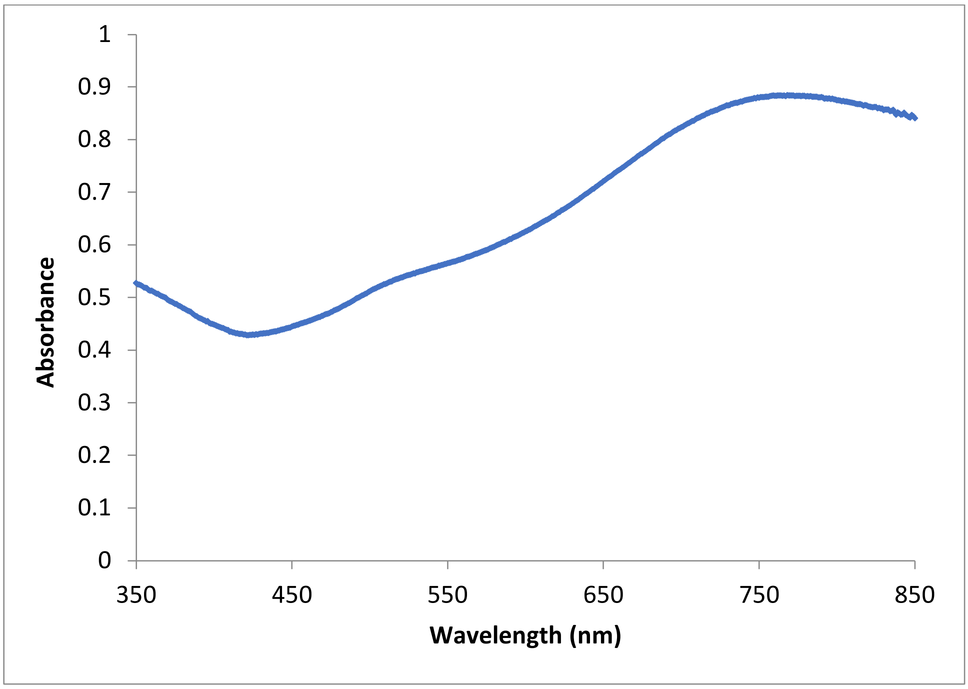

Figure S2. Typical UV-vis spectrum of a HGN suspension showing maximum absorbance around 750 - 800 nm.

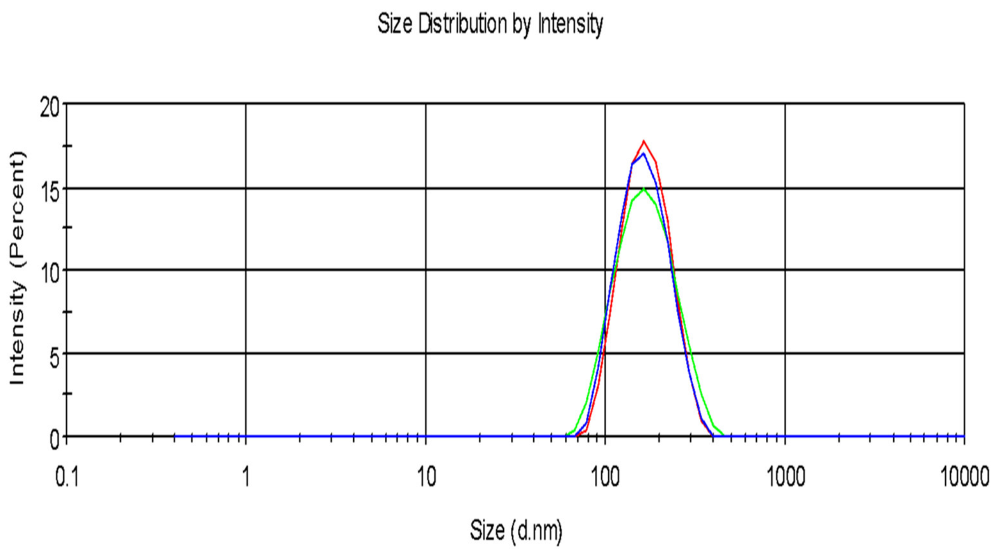

Figure S3. DLS of typical liposome suspensions indicating their size distributions.

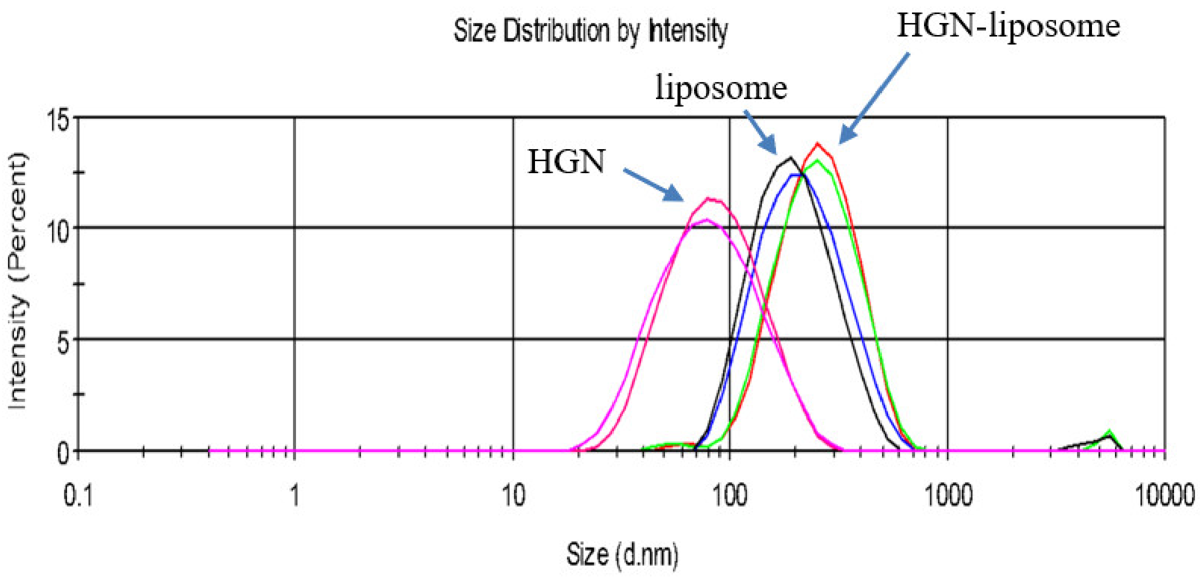

Figure S4. DLS indicating the hydrodynamic diameter of HGNs, liposomes, and HGN-liposome conjugate suspensions.

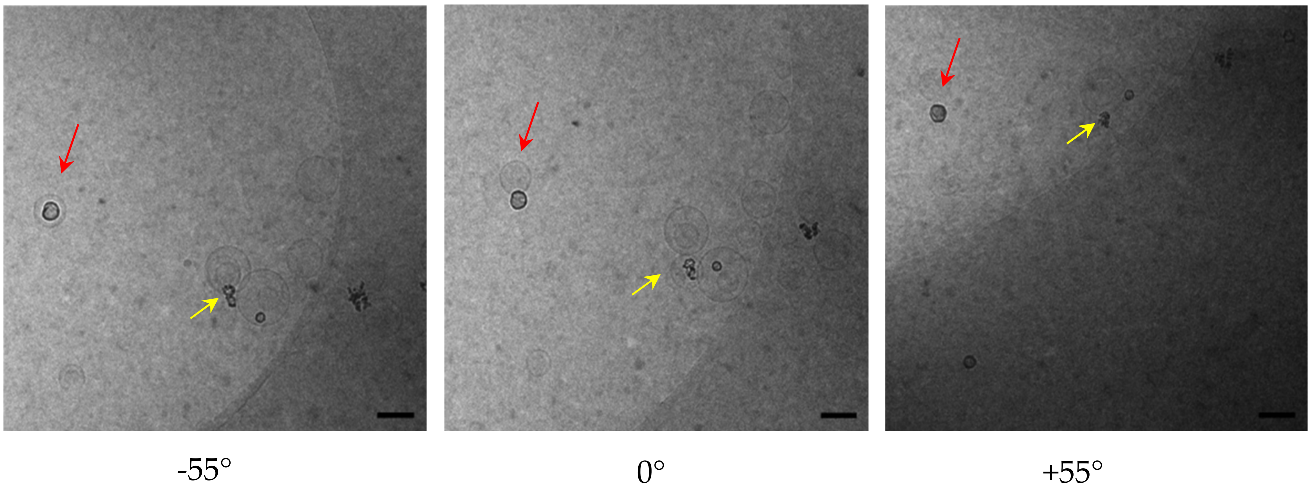

Figure S5. Cryo-TEM of HGN-liposome conjugates at different tilt angles. Scale bar is 100 nm.
